# A comparison of semi-parametric statistical modeling approaches to dynamic classification of irregularly and sparsely sampled curves

**DOI:** 10.1177/09622802251374288

**Published:** 2025-09-04

**Authors:** Ruben Deneer, Zhuozhao Zhan, Edwin Van den Heuvel, Astrid GM van Boxtel, Arjen-Kars Boer, Natal AW van Riel, Volkher Scharnhorst

**Affiliations:** 1 Department of Biomedical Engineering, 3169 Eindhoven University of Technology, Eindhoven, the Netherlands; 2 Department of Clinical Chemistry, Catharina Hospital, Eindhoven, the Netherlands; 3 Department of Mathematics and Computer science, 3169 Eindhoven University of Technology, Eindhoven, the Netherlands; 4 Department of Cardiothoracic Surgery, 3168 Catharina Hospital, Eindhoven, the Netherlands; 5 Department of Vascular Medicine, Amsterdam University Medical Center, Amsterdam, the Netherlands

**Keywords:** Longitudinal discriminant analysis, irregular sparse data, functional regression, generalized additive model

## Abstract

This study describes and compares the performance of several semi-parametric statistical modeling approaches to dynamically classify subjects into two groups, based on an irregularly and sparsely sampled curve. The motivating example of this study is the diagnosis of a complication following cardiac surgery, based on repeated measures of a single cardiac biomarker where early detection enables prompt intervention by clinicians. We first simulate data to compare the dynamic predictive performance over time for growth charts, conditional growth charts, a varying-coefficient model, a generalized functional linear model and longitudinal discriminant analysis. Our results demonstrate that functional regression approaches that implicitly incorporate historic information through random effects, provide superior discriminative ability compared to approaches that do not take historic information into account or explicitly model historic information through autoregressive terms. Semi-parametric modeling approaches show a benefit in terms of dynamic discriminative ability compared to the clinical practice of using a fixed threshold on the raw measured value. Under high degrees of sparsity the functional regression approaches are less advantageous compared to varying-coefficient models or quantile regression. The class imbalance of the outcome affects the historic and non-historic approaches in equal measure, with lower event rates reducing performance. Finally, the functional regression and varying-coefficient model were applied to a real-world clinical dataset to demonstrate their performance and application.

## 1. Introduction

Longitudinal data occurs frequently in clinical settings where patients are monitored over time. The combination of longitudinal data with a time-to-event outcome has gained popularity due to the increasing use of joint modeling techniques.^1,2^ However, in certain clinical settings, the outcome is considered *binary* instead of a *time-to-event*. In these settings, the outcome of interest is the binary status indicator that determines whether the event has occurred. There are various clinical applications for combining longitudinal data with a binary outcome. Examples include, diagnosing prostate cancer based on serial prostate-specific antigen measurements, achievement of successful pregnancy based on longitudinal measurements of lymphocyte adhesiveness, and predicting the presence of gestational trophoblastic disease based on repeated measurements of human chorionic gonadotropin.^3-5^ In literature, prediction and classification based on longitudinal data is also referred to as longitudinal discriminant analysis or longitudinal classification. Although numerous studies have explored the predictive performance of joint models with time-to-event outcomes, there is an absence of research focused on the predictive performance of various approaches to longitudinal classification.^6-9^ The aim of this study is to compare several recent semi-parametric approaches to longitudinal classification of a sparse and irregularly measured biomarker. Semi-parametric methods enable flexible modeling of frequently observed non-linear profiles in clinical settings and can be easily fitted using available software. The objective of this study is to provide guidance to researchers who are developing longitudinal classification models for sparse and irregular data. We evaluated the potential improvement in dynamic classification performance between approaches that incorporate historic information implicitly and explicitly, and more straightforward approaches that do not take historic information into account. The results are based on simulated data and supplemented with a real-world example.

This study is motivated by a real-world clinical example in which a cardiac biomarker is measured sparsely (2–5 times) and irregularly in the first 24 hours after coronary artery bypass grafting (CABG) surgery to determine the presence of a complication in the form of perioperative myocardial infarction (PMI). Detecting a PMI at an early stage enables clinicians to promptly intervene and mitigate potential harm.^10^ Therefore, the development of a model capable of dynamically classifying patients as PMI or non-PMI based on accumulating information from biomarker measurements could greatly assist clinicians in the early detection of PMI.

Longitudinal data presents an additional challenge compared to cross-sectional data, as it requires the selection of an appropriate model for the longitudinal profile. In the literature, commonly described approaches involve fitting a linear mixed-effects (LME) model to the longitudinal profile and utilizing the output of the LME model in a discriminant function.^3,11-14^ Extensions to multiple longitudinal profiles have also been developed using multivariate LME models,^15-17^ as well as non-parametric approaches in the form of functional discriminant analysis.^18,19^ Instead of employing a discriminant function for classifying individuals into groups, alternative approaches involve using summary measures derived from an LME model, such as subject-specific slopes or intercepts, as covariates in a logistic regression model. This can be achieved through a two-stage approach^20,21^ or a joint modeling approach,^4,5,22,23^ where the parameters of the mixed model and the logistic regression model are estimated simultaneously. In the two-stage approach, some researchers have employed non-parametric or non-LME models to effectively capture longitudinal trajectories.^24-27^ In addition to mixed-effects models, a more straightforward approach disregards the multilevel structure and serial correlation (i.e. historical information) of the longitudinal data. This is achieved by using a varying-coefficient model, in this case a (logistic) regression model with an interaction term between time and the covariate of interest.^28,29^ Finally, in clinical settings, reference growth charts are widely utilized as a tool to differentiate abnormal growth patterns in infants. Using specific percentile threshold values, measurements can be categorized as normal or abnormal, helping to identify atypical growth patterns.^30^ Standard reference growth charts, which do not take into account covariates or past history, are not specifically intended for screening purposes. In contrast, conditional growth charts that consider growth history are recommended as a diagnostic tool for detecting and screening unusual growth patterns.^31,32^ Although growth charts are inherently designed to detect abnormal growth, the underlying technique of quantile regression (QR) can be applied to any covariate, extending its applicability beyond growth assessment.^33^

This article is organized as follows. First, we describe the different modeling approaches that are compared. Secondly, we describe the method to simulate data from a mechanistic model and present the results of the different modeling approaches applied to the simulations. Finally, we apply the approaches to the motivating example as an illustration and show the potential clinical benefit. The article is concluded with a discussion.

## 2. Methods

We consider the situation in which a single cardiac biomarker is measured sparsely (for some patients down to one or two measurements) and irregularly up to 24 hours after surgery. During this period, a complication in the form of a PMI can occur. However, the exact time at which the complication occurs is unknown and the diagnosis is (dis)confirmed at a later time (taking multiple clinical factors into account). Therefore, the outcome is considered binary, indicating whether the patient was (at some point) diagnosed with a PMI. Our goal is to actively monitor patients for a potential PMI, based only on the accrual of information from biomarker measurements. To monitor patients, we require a *monitoring statistic* that is calculated for each patient (at each time point). The value obtained for this statistic is then used to make a decision (if the patient is at risk for a PMI) using a suitable threshold. Let $Y_{i}$ be the binary outcome (PMI confirmed yes/no) for the i -th patient, $t_{ij}$ be the time of the j -th occasion the i -th patient was measured, and $u(t_{ij})$ be the measured biomarker value at $t_{ij}$ , where $t_{ij}\in T$ , a bounded interval in R . In this study, we will compare different approaches to predict $Y_{i}$ , using information from $u(t_{ij})$ up to each $t_{ij}$ .

### 2.1. Raw value

Arguably the most straightforward approach is to use the raw value $u(t_{ij})$ itself as a monitoring statistic. This approach is currently used in the clinic, but does not take into account the time-dependent nature of $u(t_{ij})$ . If the measured biomarker concentration rises above a predefined threshold in the first 24 hours after surgery, further diagnostics are performed to confirm/rule out a PMI. In clinical practice, this threshold is based on consensus published in (international) guidelines. For this study, we used the value $u(t_{ij})$ in each $t_{ij}$ as a monitoring statistic and (after thresholding) compared it with the final diagnosis $Y_{i}$ .

### 2.2. Modeling approaches

Since we are interested in the potential benefit of different statistical modeling approaches, we use a flexible model for the longitudinal profile of $u(t_{ij})$ :

(1) $$u(t_{ij})=f(t_{ij})+\epsilon_{ij}$$

where $f(t_{ij})$ is a smooth function of the time $t_{ij}$ and $\epsilon_{ij}$ is random noise. We assume that $u(t_{ij})$ is measured on an irregular and sparse grid. In all modeling approaches, we use the generalized additive model (GAM) framework to fit the longitudinal profile $u(t_{ij})$ .^29,34^ To estimate the smooth function $f(t_{ij})$ we choose P-splines with a sufficiently large basis dimension.^35^ We can, therefore, express $u(t_{ij})$ as a set of basis functions as follows:

(2) $$u(t_{ij})=\beta_{0}+\sum_{k=1}^{K}\beta_{k}b_{k}(t_{ij})+\epsilon_{ij}$$

where $b_{k}$ are the set of K basis functions, $\beta_{0}$ is a parametric intercept term and $\beta_{k}$ the associated spline coefficients. The coefficients are estimated by maximizing the penalized log likelihood: $l_{p}(\beta )=l(\beta )-\lambda J_{\beta }$ , where $J_{\beta }$ is a penalty function based on the second-order difference of the coefficients of adjacent splines and λ a smoothing parameter that has to be chosen (for more details see Eilers and Marx).^35^ The solution to maximizing the penalized log-likelihood is obtained by penalized iteratively reweighted least squares and λ is chosen by minimizing the generalized cross-validation score, see Wood.^29^ Note that we have not yet taken dependence among observations from the same patient into account; this is deferred to the different approaches below. We use the representation in (2) for the longitudinal profile and compare the following modeling approaches to classify a new patient with at least one or more measurements: a static growth chart (SGC), a conditional growth chart (CGC), a varying-coefficient model (VCM), a generalized functional linear model (GFLM), and longitudinal discriminant analysis (LDA). The fixed cuf-off, SGC, and VCM do not take the history into account, while the CGC, LDA, and GFLM can be considered historic approaches that incorporate past measurements.

### 2.3. SGCs

SGC can be estimated through QR. QR aims at fitting the τ -th conditional quantile ( τ∈[0,1]) of u for a given $t_{ij}$ :

(3) $$Q_{u|\tau }(t_{ij})=\beta_{0\tau }+t_{ij}\beta_{1\tau }+\epsilon_{ij,\tau }$$

where $\beta_{0\tau }$ is the intercept belonging to the τ -th quantile and $\beta_{1\tau }$ is the coefficient belonging to the τ -th quantile and $\epsilon_{ij,\tau }$ random noise belonging to the τ -th quantile. In (3), the assumption is made that the τ -th quantile depends linearly on the covariate $t_{ij}$ . Fasiolo et al.^36^ have developed a novel framework that combines QR with a GAM, resulting in a quantile GAM (QGAM). The advantage of this framework is that, aside from incorporating smooth functions, all smoothing and hyperparameters are estimated automatically. Using the QGAM framework, the conditional quantile τ of u is given by the following equation:

(4) $$Q_{u|\tau }(t_{ij})=f_{\tau }(t_{ij})+\epsilon_{ij,\tau }$$

where $f_{\tau }(t_{ij})$ is smoothing function represented by a P-spline and $\epsilon_{ij,\tau }$ is the residual error term. Parameter estimates of the τ th conditional quantile are obtained by minimizing the expected loss:

(5) $$\begin{array}{rl} L(f_{\tau }|t_{ij}) & =\sum_{i=1}^{n}\sum_{j=1}^{m}\rho_{\tau }(u(t_{ij})-f_{\tau }(t_{ij}))\\ \rho_{\tau }(z) & =(\tau -1)\frac{z}{\sigma }+\lambda \text{log}(1+e^{\frac{z}{\lambda \sigma }}) \end{array}$$

$\rho_{\tau }(z)$ is the so-called extended log-f loss, σ is a scale parameter, and λ is a penalty factor that determines the smoothness of the loss. By using the fast calibrated Bayesian methods proposed by Fasiolo et al., QGAMs can be fitted with this loss function. $Q_{u|\tau }(t_{ij})$ represents the value of the τ -th quantile of $u(t_{ij})$ and we fit (5) for a vector of quantiles τ=(0.01,0.02,…,0.99) to data of control patients (i.e. patients that were not diagnosed with a PMI). Thus, we obtain a reference growth chart for a suitable grid of quantiles which can then be used to dynamically classify new patients depending on the measured $u(t_{ij})$ and a chosen quantile above which measurements are considered positive for a PMI. That is, the monitoring statistic of this approach is the assigned quantile of the measured $u(t_{ij})$ .

### 2.4. CGCs

In this study, we implement the CGC as described by Wei et al.^32^ in the QGAM framework of Fasiolo et al.^36^ We expand (4) as follows:

(6) $$Q_{cond,u|\tau }(t_{ij})=f_{\tau }(t_{ij})+\sum_{k=1}^{p}(\alpha_{k,\tau }+\theta_{k,\tau }D_{i,j,k})t_{i,j-k}+\epsilon_{ij,\tau }$$

where $D_{i,j,k}=t_{i,j}-t_{i,j-k}$ is the time between the j -th and (j−k) -th measurement, and $\alpha_{k,\tau }$ and $\theta_{k,\tau }$ are parametric autoregressive (AR) coefficients. We choose p=1 , that is, an AR(1) model. Analogous to the SGC approach, the model is fitted only to control patients and, new measurements can be classified as positive or negative for a PMI, depending on a chosen threshold quantile.

### 2.5. VCM

An alternative to the growth chart approach that directly models the probability of the outcome $Y_{i}$ , rather than estimating quantiles of a healthy population, is the VCM. In the case of a VCM, we directly predict the probability of having the outcome, $P(Y_{i}=1|u(t_{ij}),t_{ij})$ , for a measurement pair $\left(u(t_{ij}),t_{ij}\right)$ . Essentially, this a logistic regression model with an interaction between time and the measured value as covariate, also called a varying-coefficient model. For this, we use a GAM with a logistic link function and a tensor product smooth interaction (as $u(t_{ij})$ and $t_{ij}$ are on different scales):

(7) $$\text{logit}\{P(Y_{i}=1|u(t_{ij}),t_{ij})\}=f(u(t_{ij}),t_{ij})=\sum_{k=1}^{K}\sum_{l=1}^{L}\beta_{kl}b_{l}(u(t_{ij}))a_{k}(t_{ij})$$

where $a_{k}$ and $b_{l}$ are sets of P-spline basis functions for $t_{ij}$ and $u(t_{ij})$ , respectively. The predicted probability $P(Y_{i}=1|u(t_{ij}),t_{ij})$ is used to classify patients as positive or negative. This is analogous to the growth chart approach, except that in this approach the predicted probability (rather than the quantile) is the monitoring statistic for which a threshold has to be chosen above which patients are considered positive.

### 2.6. GFLM

The GFLM is described by Müller^24^ for observations observed on dense grids of points.^24^ The general idea is to reduce the dimension of the longitudinal data by an orthogonal expansion of the random effects and use the first few components of the expansion as covariates in a generalized linear model, for example, logistic regression model. This procedure can also be applied to our study, with some modification as observations are irregularly and sparsely observed. We model $u(t_{ij})$ as follows:

(8) $$\begin{array}{rl} u(t_{ij}) & =f(t_{ij})+U_{i}(t_{ij})+\epsilon_{ij}\\ & =\beta_{0}+\sum_{k=1}^{K}\beta_{k}b_{k}(t_{ij})+U_{i}(t_{ij})+\epsilon_{ij}\\ U_{i}(t_{ij}),U_{i}(t_{ij}^{\prime }) & ∼N(0,\Sigma (t_{ij},t_{ij}^{\prime })),\epsilon_{ij}∼N(0,\sigma^{2}) \end{array}$$

where $\beta_{0}$ is a parametric intercept, $b_{k}$ are sets of P-spline basis functions with coefficients $\beta_{k}$ and dimension K , $U_{i}(t_{ij})$ are functional random effects representing the subject specific deviation from the overall mean function, modeled as a zero-mean Gaussian process with variance-covariance function $\Sigma (t_{ij},t_{ij}^{\prime })$ . More specifically, $\Sigma (t_{ij},t_{ij}^{\prime })=\text{cov}(U_{i}(t_{ij}),U_{i}(t_{ij}^{\prime }))$ . Since we are dealing with irregular and sparse data, the estimation of the covariance function is not as straightforward as with a suitably dense grid. Therefore we use the fast covariance estimation for sparse functional data (FACEs) approach by Xiao et al.^37^ In this approach, the covariance function $\Sigma (t_{ij},t_{ij}^{\prime })$ is modeled by penalized tensor product smooths $\Sigma (t_{ij},t_{ij}^{\prime })=b(t_{ij}{)}^{⊺}\Theta b(t_{ij}^{\prime })$ , where b is a spline basis and $\Theta =(\theta_{k,l}{)}_{1\leq k\leq c,1\leq l\leq c}$ a symmetric coefficient matrix with c equal to the degrees of freedom of the spline basis. The covariance function and error variance are jointly estimated in a two-step procedure and smoothing parameters are selected using leave-one-subject out. For more details see Xiao et al.^37^ The covariance function $\Sigma (t_{ij},t_{ij}^{\prime })$ can be decomposed into functional principal components: $\Sigma (t_{ij},t_{ij}^{\prime })=\sum_{l=1}^{\infty }\lambda_{l}\phi_{l}(t_{ij})\phi_{l}(t_{ij}^{\prime })$ , where $\lambda_{l}$ and $\phi_{l}(t_{ij})$ are the respective eigenvalues and eigenfunctions. We choose a number of eigenfunctions L that explain 95% of total variance. By the Karhunen-Loève theorem we can project $U_{i}(t_{ij})$ onto the L -dimensional basis, and (8) can be written as follows:

(9) $$\begin{array}{rl} u(t_{ij})= & \beta_{0}+\sum_{k=1}^{K}\beta_{k}b_{k}(t_{ij})+\sum_{l=1}^{L}\xi_{i,l}\phi_{l}(t_{ij})+\epsilon_{ij}\\ & \xi_{i,l}∼N(0,\lambda_{l}),\epsilon_{ij}∼N(0,\sigma^{2}) \end{array}$$

Since data are sparse and irregular, the scores $\xi_{i,l}$ are estimated by the principal components analysis through conditional expectation approach described by Yao et al.^38^ After estimating all scores for the subjects in the dataset, a logistic regression model is fitted with the scores $\xi_{i,l}$ as covariates and $Y_{i}$ as outcome. If we wish to make a prediction for a new subject, we first estimate the scores $\xi_{i,l}$ by using the conditional expectation and subsequently plug the scores in the logistic regression model to obtain the probability that the patient has a PMI:

(10) $$\text{logit}\{P(Y_{i}=1|\xi_{i,1},\xi_{i,2},\ldots ,\xi_{i,L})\}=\sum_{l=1}^{L}\gamma_{l}\xi_{i,l}$$

where $\gamma_{l}$ are the coefficients belonging to the L scores. The predicted probabilities are then used as monitoring statistic to classify patients as positive or negative for a PMI.

### 2.7. LDA

The LDA approach consists of two steps. In the first step, the inherently infinite-dimensional curves are projected onto a low-dimensional space, in the second step, the low-dimensional representation is used to perform discriminant analysis. In this study, we implement both a covariance pattern longitudinal discriminant analysis (COV-LDA) as described by Roy et al.^39^ and a functional linear discriminant analysis (F-LDA) as described by James and Hastie.^18^

#### 2.7.1. COV-LDA

The COV-LDA model consists of a linear additive model with a parametric intercept term, a factor smooth interaction term, and a covariance pattern (i.e. correlation structure) to model dependence among observations within a single patient. The factor smooth interaction term allows for separate smooths for both PMI and non-PMI patients:

(11) $$\begin{array}{rl} u(t_{ij}) & =\beta_{0}+z_{i}\beta_{1}+f_{z_{i}}(t_{ij})+\epsilon_{ij}\\ & =\beta_{0}+z_{i}\beta_{1}+\sum_{k=2}^{K}\beta_{z_{i},k}b_{z_{i},k}(t_{ij})+\epsilon_{ij},\\ \epsilon_{i} & =\left[\begin{array}{c} \epsilon_{i1}\\ \epsilon_{i2}\\ \vdots \\ \epsilon_{im} \end{array}\right]∼N(0,\sigma^{2}\Lambda_{i})\\ z_{i} & =\left\{ \begin{array}{ll} 0, & \text{if patient}\;i\;\text{was not diagnosed with a PMI}\\ 1, & \text{if patient}\;i\;\text{was diagnosed with a PMI} \end{array}\right. \end{array}$$

where $\beta_{0}$ is a parametric intercept, $\beta_{1}$ is a class-specific parametric effect, $b_{z_{i},k}$ are sets of P-spline basis functions with coefficients $\beta_{z_{i},k}$ , and dimension K for non-PMI and PMI patients, respectively, and $\Lambda_{i}$ is a covariance matrix. $\Lambda_{i}$ can be decomposed, $\Lambda_{i}=V_{i}C_{i}V_{i}$ , where $V_{i}$ is a diagonal, and $C_{i}$ a correlation matrix. Since observations are irregularly sampled, we choose a continuous-time AR(1) correlation structure for $C_{i}$ to model the dependence between measurements of the same subject.^40^

#### 2.7.2. F-LDA

The alternative F-LDA approach does not assume a correlation structure for the residual error but utilizes random effects to capture variability between patients. In the F-LDA approach, we model the profile $u(t_{ij})$ as follows:

(12) $$\begin{array}{rl} u(t_{ij}) & =\beta_{0}+z_{i}\beta_{1}+f_{z_{i}}(t_{ij})+U_{i}(t_{ij})+\epsilon_{ij}\\ & =\beta_{0}+z_{i}\beta_{1}+\sum_{k=2}^{K}\beta_{z_{i},k}b_{z_{i},k}(t_{ij})+U_{i}(t_{ij})+\epsilon_{ij}\\ U_{i}(t_{ij}),U_{i}(t_{ij}^{\prime }) & ∼N(0,\Sigma (t_{ij},t_{ij}^{\prime })),\epsilon_{ij}∼N(0,\sigma^{2})\\ z_{i} & =\left\{ \begin{array}{ll} 0, & \text{if patient}\;i\;\text{was not diagnosed with a PMI}\\ 1, & \text{if patient}\;i\;\text{was diagnosed with a PMI} \end{array}\right. \end{array}$$

where $\beta_{0}$ , $\beta_{1}$ , and $b_{z_{i},k}$ are analogous to (11) and $U_{i}(t_{ij})$ are (functional) random effects with variance-covariance function $\Sigma (t_{ij},t_{ij}^{\prime })$ analogous to (8). Also analogous to (8), we use the FACEs procedure by Xiao et al.^37^ to estimate the smoothed covariance matrix from the sparse data.

After fitting (11) and (12), we can obtain estimates for a new subject given a set of measurement times $t_{ij}$ . By plugging $t_{ij}$ in either (11) or (12), we obtain estimates for the mean if the patient would belong to the PMI group, $\hat{u}(t_{ij},z_{i}=1)$ , or the non-PMI group $\hat{u}(t_{ij},z_{i}=0)$ and a covariance matrix (either by imposing a correlation structure in (11) or by modeling the covariance matrix in (12)). Given the observed set of measurements $u(t_{ij})$ for the new subject, we can then calculate the value of the probability density function in the case the patient belongs to the PMI or to the non-PMI group. These values can then be utilized in a Bayes discriminant rule to obtain the probability of a PMI:

(13) $$P(Y_{i}=1|u(t_{ij}),t_{ij})=\frac{\pi_{\text{PMI}}f_{\text{PMI}}(u(t_{ij}))}{\pi_{\text{no-PMI}}f_{\text{no-PMI}}(u(t_{ij}))+\pi_{\text{PMI}}f_{\text{PMI}}(u(t_{ij}))}$$

where $\pi_{\text{PMI}}$ is the prior probability of having a PMI and $\pi_{\text{no-PMI}}=1-\pi_{\text{PMI}}$ , $f_{\text{PMI}}$ is the conditional density function if the patient had a PMI and $f_{\text{no-PMI}}$ is the conditional density function if the patient did not have a PMI. The prior probabilities $\pi_{\text{PMI}}$ and $\pi_{\text{no-PMI}}$ are equal to the fraction of non-PMI and PMI patients in the dataset, respectively. The probability $P(Y_{i}=1|u(t_{ij}))$ is then used as monitoring statistic to classify patients as positive or negative, analogous to the previous approaches.

## 3. Simulations

To compare the different approaches, we simulate data based on a biexponential (pharmacokinetic) model that reflects the release of a cardiac biomarker after surgery and clearance from the circulation by the kidneys; see the following equation:

(14) $$\begin{array}{rl} c_{i}(t_{j}) & =\phi_{1i}e^{-\phi_{3i}t_{j}}+\phi_{2i}e^{-\phi_{4i}t_{j}}+\epsilon_{ij}\\ \phi_{i} & =\left[\begin{array}{c} \phi_{1i}\\ \phi_{2i}\\ \phi_{3i}\\ \phi_{4i} \end{array}\right]=\left[\begin{array}{c} \beta_{1}\\ \beta_{2}\\ \beta_{3}\\ \beta_{4} \end{array}\right]+\left[\begin{array}{c} \gamma_{1}z_{i}\\ \gamma_{2}z_{i}\\ \gamma_{3}z_{i}\\ \gamma_{4}z_{i} \end{array}\right]+\left[\begin{array}{c} b_{1i}\\ b_{2i}\\ b_{3i}\\ b_{4i} \end{array}\right]=\beta +\gamma z_{i}+b_{i}\\ z_{i} & =\left\{ \begin{array}{ll} 0, & \text{if patient}\;i\;\text{was not diagnosed with a PMI}\\ 1, & \text{if patient}\;i\;\text{was diagnosed with a PMI} \end{array}\right.\\ b_{i} & ∼N(0,\Psi ),\;\epsilon_{ij}∼N(0,\sigma^{2}) \end{array}$$

where β and γ are the fixed effects, $b_{i}$ are the random effects with covariance matrix Ψ . Parameter values for β , γ , Ψ , and $\sigma^{2}$ are given in (15) and were obtained by fitting (14) to clinical trial data from patients who underwent CABG surgery.

(15) $$\begin{array}{rl} \phi_{i} & =\left[\begin{array}{c} -1.59-0.233z_{i}\\ \;2.73+0.19z_{i}\\ 0.457-0.0903z_{i}\\ 0.00928-0.0118z_{i} \end{array}\right]\\ \Psi & =\left[\begin{array}{cccc} 0.1770 & -0.0786 & -0.0218 & 0\\ -0.0786 & 0.1450 & -0.0233 & 0\\ -0.0218 & -0.0233 & 0.0357 & 0\\ 0 & 0 & 0 & 2.36\times 10^{-6} \end{array}\right]\\ \sigma^{2} & =0.0159 \end{array}$$

**Figure 1.**
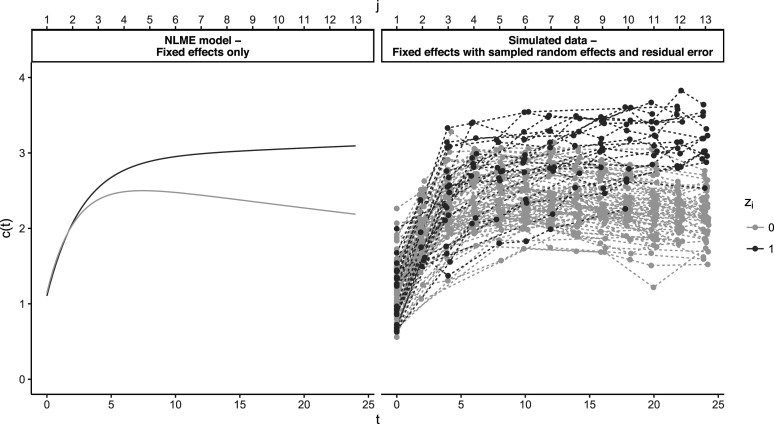
Plot of simulated data from the non-linear mixed effects (NLMEs) bi-exponential model. Left: marginal/fixed effects for perioperative myocardial infarction (PMI) and non-PMI patients. Right: fixed effects combined with sampled subject-specific effects and residual error. *N* = 100 patients were sampled with an event rate of 20%, that is, 80 did not experience a PMI ( $z_{i}=0$ ) and 20 patients did ( $z_{i}=1$ ). The degree of sparsity ( $P_{\mathrm{miss}}$ ) was set to 50%.

For each simulated patient i , we start with the sequence $t_{j}=\{0,2,4,6,8,12,16,20,24\}$ . First, irregularity in measurement times is introduced by adding variation to each $t_{j>1}$ by sampling from a uniform distribution between − 0.25 and 0.25. Second, this irregular sequence $t_{j}$ is used in the bi-exponential model (14) to obtain simulated $c_{i}(t_{j})$ . Fixed effects are given in (15), random effects are sampled from a multivariate normal distribution with covariance matrix Ψ and residual error is sampled from a normal distribution with variance $\sigma^{2}$ . If, as a result of random sampling, any $c_{i}(t_{j})\leq 0$ , the sampling of random effects for that patient is repeated until all $c_{i}(t_{j})>0$ . Finally, sparsity is introduced by randomly removing elements $t_{j>1}$ with a (chosen) probability $P_{\mathrm{miss}}$ . In Figure 1, an example of simulations generated by the model is visualized. As described previously, training and test sets are required to objectively evaluate performance. We generate 50 training and 50 test sets, each set containing N=500 patients. We vary the degree of sparsity (by choosing different fractions of missingness ( $P_{\mathrm{miss}}$ )) and the event rate of the outcome.

### 3.1. Classification and performance evaluation

Since the goal of this study is to perform dynamic longitudinal classification, we focus on the performance of the approaches in a dynamic fashion. We generate separate training and test sets and fit all the approaches to the training sets. Then, we evaluate the time-dependent area under the ROC curve (AUC) by calculating the AUC at each j -th time point on the test set. At each measurement occasion j , we use the monitoring statistic of the specific approach as input for the ROC curve and the binary diagnosis $Y_{i}$ as outcome. The procedure is repeated for all approaches to obtain the time-dependent AUC for each approach. Second, we evaluate the AUC of the maximum value of the monitoring statistic in the time interval T , we refer to this as the ‘‘dynamic classification” AUC. As a rationale, we refer to the clinical practice, where, if the biomarker exceeds a certain threshold, the patient is classified as positive and further diagnostics will take place. On the first occasion $t_{ij}$ , the monitoring statistic exceeds a threshold, the patient is classified as positive (even if the monitoring statistic at $t_{ij+1}$ falls below the threshold). Thus, given a threshold, the maximum in the time interval T determines if a patient is classified as positive or negative. Finally, the ROC curves of the maxima are used to define a threshold for each approach based on the Youden index. This threshold is then used as an early stopping rule, we classify a patient as positive if the monitoring statistic rises above this threshold and mark the time that this occurs. We then calculate the sensitivity, specificity and average run length (ARL) of each approach using the stopping rule. The ARL represents the mean time until a patient is classified as positive. The ARL is based on true positives.

## 4. Implementation

All approaches are implemented using R version 4.4.1.^41^ The P-spline smooths are modeled using the s function from mgcv package version 1.9-1. The SGC and CGC are fitted using the qgam package version 1.3.4. The VCM is fitted using gam and te functions from mgcv. To fit the COV-LDA model, the gamm function is used in conjunction with the corCAR1 function from the nlme package as a constructor for the correlation matrix, parameters in the COV-LDA model are estimated using restricted maximum likelihood. To estimate the covariance function in the F-LDA model and GFLM, we use the face.sparse function from the face package version 0.1-7. The densities of the normal distributions, required by the Bayes rule, are calculated using the dmvnorm function in the mvtnorm package version 1.2-5. In simulations, sampling from the multivariate normal distribution is performed using the mvrnorm function in the MASS package. The code to fit and predict from the different approaches is provided as the Supplemental Material.

## 5. Results

**Figure 2.**
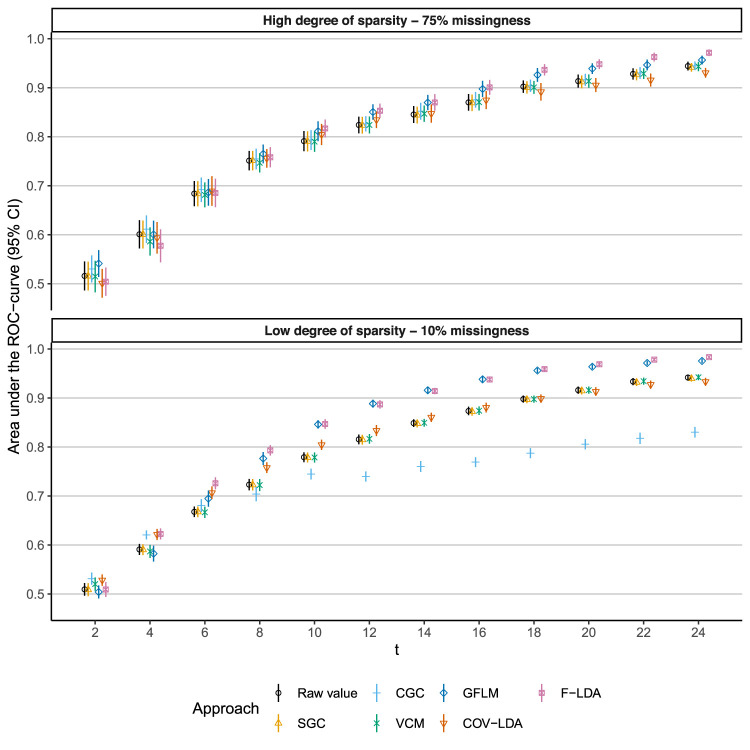
Time-dependent area under the ROC curve (AUC) under high and low degrees of sparsity. Each approach was fitted on a training set and performance was evaluated in a time-dependent fashion on a test set. This process was repeated 50 times to obtain means and standard errors. SGCs: static growth charts; CGCs: conditional growth charts; VCMs: varying-coefficient models; COV-LDA: covariance pattern longitudinal discriminant analysis; F-LDA: functional longitudinal discriminant analysis; GFLM: generalized functional linear model.

**Figure 3.**
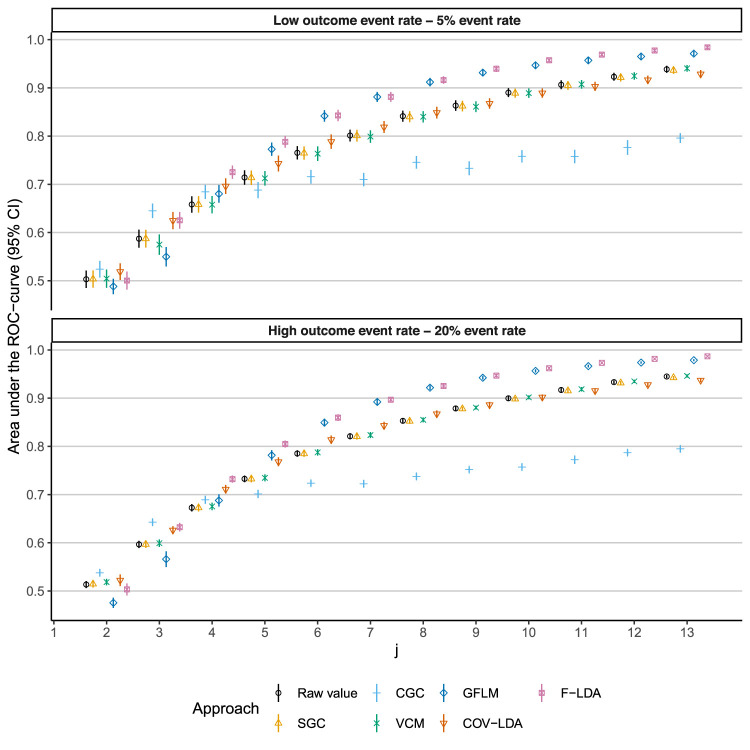
Time-dependent area under the ROC curve (AUC) under low and high outcome (PMI) event rates. Each approach was fitted on a training set and performance was evaluated in a time-dependent fashion on a test set. This process was repeated 50 times to obtain means and standard errors. PMI: perioperative myocardial infarction; SGCs: static growth charts; CGCs: conditional growth charts; VCMs: varying-coefficient models; COV-LDA: covariance pattern longitudinal discriminant analysis; F-LDA: functional longitudinal discriminant analysis; GFLM: generalized functional linear model.

The time-dependent AUC is plotted over time for different degrees of sparsity in Figure 2 and for different event rates in Figure 3. Numerical values for different degrees of sparsity/event rates can be found in the Supplemental Material. The functional regression approaches (GFLM and FLDA) show a clear benefit in discriminative ability starting from t=8 , except for the setting with a high degree of sparsity where the benefit of these approaches becomes apparent at t=18 . For a high degree of sparsity (75% missingness) each subject has, on average, four longitudinal measurements, compared to an average of 12 measurements for the low degree of sparsity (10% missingness).

The dynamic classification AUC is reported in Tables 1 and 2 for different degrees of sparsity and event rates, respectively. This table reflects the performance when taking the maximum value of the monitoring statistic for each patient in the time interval T . The F-LDA and GFLM are clearly superior in terms of dynamic discriminative ability, although this benefit is less pronounced in the setting with a high degree of sparsity.

**Table 1.** The AUC with standard error in round brackets, when using each approach to dynamically classify new patients under a high degree of sparsity (75% missingness) and a low degree of sparsity (10% missingness).

Dynamic classification AUC		
	High degree of sparsity	Low degree of sparsity
Raw value	0.846 (0.005)	0.864 (0.004)
SGC	0.860 (0.005)	0.900 (0.004)
CGC	0.855 (0.005)	0.859 (0.004)
VCM	0.849 (0.005)	0.893 (0.005)
GFLM	0.886 (0.006)	0.958 (0.003)
COV-LDA	0.839 (0.005)	0.873 (0.006)
F-LDA	0.875 (0.005)	0.956 (0.004)

Each approach was fitted on a training set and performance was evaluated on a test set. This process was repeated 50 times to obtain means and standard errors. AUC: area under the ROC curve; SGCs: static growth charts; CGCs: conditional growth charts; VCMs: varying-coefficient models; COV-LDA: covariance pattern longitudinal discriminant analysis; F-LDA: functional longitudinal discriminant analysis; GFLM: generalized functional linear model.

**Table 2.** The AUC with standard error in round brackets, when using each approach to dynamically classify new patients under a low event rate (5%) and a high event rate (20%).

Dynamic classification AUC		
	Low outcome event rate	High outcome event rate
Raw value	0.857 (0.005)	0.869 (0.002)
SGC	0.892 (0.004)	0.903 (0.002)
CGC	0.830 (0.005)	0.839 (0.003)
VCM	0.885 (0.006)	0.901 (0.003)
GFLM	0.955 (0.003)	0.945 (0.002)
COV-LDA	0.863 (0.008)	0.879 (0.004)
F-LDA	0.953 (0.004)	0.961 (0.002)

Each approach was fitted on a training set and performance was evaluated on a test set. This process was repeated 50 times to obtain means and standard errors. AUC: area under the ROC curve; SGCs: static growth charts; CGCs: conditional growth charts; VCMs: varying-coefficient models; COV-LDA: covariance pattern longitudinal discriminant analysis; F-LDA: functional longitudinal discriminant analysis; GFLM: generalized functional linear model.

In Tables 3 and 4, the sensitivity, specificity, threshold, and ARL of each approach is given under varying degrees of sparsity and outcome event rates. The threshold is based on the Youden index. The F-LDA and GFLM approaches show the best results in terms of combining a high sensitivity, specificity, but at the expense of a somewhat longer ARL than the raw value.

**Table 3.** Sensitivity, specificity threshold, and ARL in hours with standard error in round brackets, for each approach for high and low degrees of sparsity.

High degree of sparsity—75% missingness							
	Raw value	SGC	CGC	VCM	GFLM	COV-LDA	F-LDA
Sensitivity	0.824 (0.009)	0.828 (0.010)	0.823 (0.011)	0.871 (0.008)	0.870 (0.008)	0.870 (0.009)	0.894 (0.007)
Specificity	0.763 (0.010)	0.781 (0.011)	0.780 (0.011)	0.743 (0.010)	0.792 (0.011)	0.717 (0.011)	0.768 (0.010)
Threshold	2.803 (0.013)	89.560 (0.735)	91.380 (0.626)	0.200 (0.010)	0.181 (0.009)	0.232 (0.011)	0.221 (0.008)
ARL	12.610 (0.143)	13.618 (0.180)	13.522 (0.181)	13.961 (0.202)	14.566 (0.258)	13.052 (0.236)	14.146 (0.213)
Low degree of sparsity—10% missingness							
	Raw value	SGC	CGC	VCM	GFLM	COV-LDA	F-LDA
Sensitivity	0.827 (0.010)	0.859 (0.007)	0.799 (0.011)	0.900 (0.006)	0.916 (0.005)	0.914 (0.006)	0.948 (0.004)
Specificity	0.784 (0.011)	0.832 (0.009)	0.802 (0.011)	0.806 (0.008)	0.909 (0.007)	0.762 (0.010)	0.896 (0.007)
Threshold	2.934 (0.012)	95.820 (0.312)	97.780 (0.154)	0.325 (0.013)	0.247 (0.016)	0.379 (0.016)	0.494 (0.017)
ARL	10.618 (0.127)	12.936 (0.215)	12.785 (0.166)	13.942 (0.180)	13.416 (0.185)	12.769 (0.241)	14.373 (0.173)

A threshold based on the Youden index was used as a stopping rule. That is, if, for a patient, the monitoring statistic of an approach rises above this threshold, the patient is classified as positive. Note that the monitoring statistic represents a value in case of the raw value, a quantile in case of the SCG and CGC approaches and a probability in the VCM, GFLM, COV-LDA, and F-LDA approaches. The mean time until a positive classification is represented by the ARL. ARL: average run length; SGCs: static growth charts; CGCs: conditional growth charts; VCMs: varying-coefficient models; COV-LDA: covariance pattern longitudinal discriminant analysis; F-LDA: functional longitudinal discriminant analysis; GFLM: generalized functional linear model.

**Table 4.** Sensitivity, specificity threshold, and average run length (ARL) in hours with standard error in round brackets, for each approach for low and high event rates.

Low event rate—5% event rate							
	Raw value	SGC	CGC	VCM	GFLM	COV-LDA	F-LDA
Sensitivity	0.826 (0.012)	0.853 (0.010)	0.756 (0.013)	0.899 (0.008)	0.916 (0.006)	0.904 (0.009)	0.947 (0.004)
Specificity	0.787 (0.013)	0.838 (0.011)	0.791 (0.015)	0.812 (0.012)	0.910 (0.007)	0.759 (0.015)	0.897 (0.007)
Threshold	2.945 (0.016)	95.540 (0.467)	97.720 (0.157)	0.207 (0.013)	0.132 (0.011)	0.258 (0.019)	0.390 (0.025)
ARL	10.656 (0.196)	12.710 (0.222)	11.711 (0.226)	14.089 (0.234)	13.536 (0.165)	12.815 (0.277)	14.467 (0.264)
High event rate—20% event rate							
	Raw value	SGC	CGC	VCM	GFLM	COV-LDA	F-LDA
Sensitivity	0.848 (0.006)	0.886 (0.006)	0.744 (0.010)	0.909 (0.006)	0.894 (0.005)	0.926 (0.004)	0.950 (0.003)
Specificity	0.763 (0.008)	0.808 (0.008)	0.809 (0.010)	0.803 (0.008)	0.880 (0.006)	0.759 (0.008)	0.904 (0.004)
Threshold	2.958 (0.009)	96.960 (0.232)	97.540 (0.118)	0.528 (0.013)	0.530 (0.016)	0.566 (0.012)	0.648 (0.014)
ARL	10.397 (0.112)	12.855 (0.174)	11.359 (0.139)	13.465 (0.180)	11.627 (0.126)	12.608 (0.184)	13.664 (0.158)

A threshold based on the Youden index was used as a stopping rule. That is, if for a patient the monitoring statistic of an approach rises above this threshold, the patient is classified as positive. Note that the monitoring statistic represents a value in case of the raw value, a quantile in case of the SGC and CGC approaches and a probability in the VCM, GFLM, COV-LDA, and F-LDA approaches. The mean time until a positive classification is represented by the ARL. ARL: average run length; SGCs: static growth charts; CGCs: conditional growth charts; VCMs: varying-coefficient models; COV-LDA: covariance pattern longitudinal discriminant analysis; F-LDA: functional longitudinal discriminant analysis; GFLM: generalized functional linear model.

## 6. Clinical case study: PMI after CABG surgery

**Figure 4.**
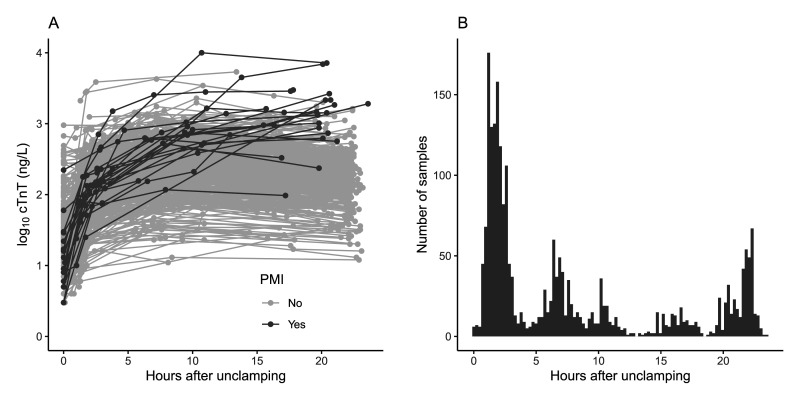
Overview of studydata. (a) Spaghetti plot of time after aortic unclamping against the $\log_{10}$ transformed value of the measured cardiac troponin (cTn)T concentration in ng/L. A total of 2892 cTnT values were measured for 639 patients undergoing coronary artery bypass grafting surgery. Profiles of patients diagnosed with a perioperative myocardial infarction (PMI) ( N= 22) are shown in dark gray, patients without PMI in light gray. (b) Histogram of sampling times (excluding t=0 ). cTnT was measured at t= 0 (before surgery) and at irregular times after surgery, centering around 1.5, 2, 6, 12, and 24 hours after aortic unclamping.

**Figure 5.**
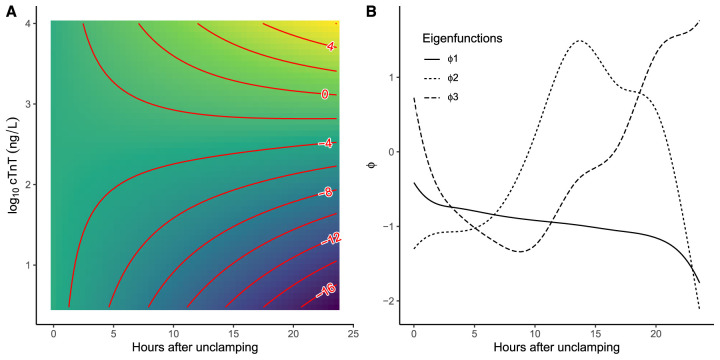
Predictions from the varying-coefficient model (VCM) and generalized functional linear model (GFLM) approaches. (a) Contour plot with contour lines in red, reflecting the predicted probability of a perioperative myocardial infarction (PMI) by the VCM approach. Note that the predictions are on the linear predictor scale which can be converted to a probability by applying the logit function. For example, the “0” contour line, represents the line with a probability of a PMI of 0.5. (b) This plot shows the three eigenfunctions that explain 95 % of the variance, extracted by the FACEs approach of the GFLM. The first eigenfunction ϕ1 is negatively associated with the outcome of a PMI, whereas the second eigenfunction ϕ2 is positively associated with a PMI. By obtaining conditional expectations for a new subject, based on these eigenfunctions, the probability of a PMI can be obtained.

As described in Section 1, this study is motivated by the need to detect patients who experience PMI after having undergone CABG surgery, based on serial measurements of a cardiac biomarker. After surgery, cardiac biomarkers are repeatedly sampled in patients to detect a possible PMI. A PMI is defined as a procedural myocardial infarction whose pathogenesis is multifactorial and can be graft-related or non-graft-related.^42,43^ Examples of graft-related PMI include graft failure due to occlusion, kinking, or overstretching. Non-graft related PMI can result from procedural difficulties like trauma from surgical manipulation or inadequate myocardial protection. The post-operative rise of cardiac biomarkers, in particular cardiac troponin (cTn), can reflect myocardial damage originating from either (early) graft failure or non-graft related causes. In the former case, minimizing the time to a re-intervention is crucial to save viable myocardium. However, all patients experience an unavoidable increase in cardiac biomarkers, simply as a result of the procedure itself. For this study, a dataset of 639 patients who underwent CABG surgery at Catharina Hospital in Eindhoven, The Netherlands, is available. For more details on the study, see Deneer et al.^44^ For each patient, cTnT was sampled up to 24 hours after surgery and the outcome (PMI yes/no) was recorded. Sampling of cTnT was irregular and more frequent in the first 6 hours after surgery, see Figure 4(b). Patients with a PMI generally show a sustained release of cTnT from damaged myocardium, instead of a rising-and-falling trend in the first 24 hours after surgery,^44-46^ see Figure 4(a). As this is a clinical case study and there are only a small number of cases, leave-one-subject-out cross-validation was used to estimate performance. To calculate the time-dependent AUC, only patients who had at least two measurements before t=6 and at least one measurement after t=12 were included, resulting in 520 patients being included, of which 21 had a PMI. All approaches were fitted to this dataset. Visualizations of the VCM and GFLM can be seen in Figure 5. As the data are too irregularly sampled to calculate the AUC on an hourly basis, the AUC was calculated at t=6,12 , and 24 hours after surgery, again using the cumulative maximum as previously described. Tables 5 and 6 show the AUC of cTnT versus the different non-historic and historic approaches, respectively. The dynamic classification AUC is given in Table 7. To determine whether there is a clinical benefit in using a model-based approach instead of the raw biomarker value as a threshold to initiate a further diagnosis of a PMI, we compared model-based approaches with the current clinical guideline in terms of sensitivity, specificity, and ARL. Since the clinical guideline recommends a threshold of 140 ng/L for cTnT,^43^ in the study, dataset this corresponds to a sensitivity of 0.952. Using ROC curve analysis, we calculated a threshold for each approach corresponding to a sensitivity of 0.952. Subsequently, the performance in terms of specificity, true positives, false positives, true negatives, false negatives, and ARL was compared; see Table 8. We conclude that the modeling-based approaches can provide a similar sensitivity as the guideline, whilst offering a higher specificity which greatly reduces the number of false positives, at the expense of a longer time until detection.

**Table 5.** The area under the ROC curve (AUC) and the 95% confidence interval for each of the non-historic approaches applied to the study dataset using leave-one-subject-out cross-validation and information up to time t .

	cTnT	SGC	CGC	VCM
≤6 hours	0.560 ( 0.432−0.689 )	0.496 (0.364−0.627)	0.609 (0.482−0.736)	0.431 (0.291−0.571)
≤12 hours	0.712 (0.577−0.847)	0.646 (0.522−0.771)	0.719 (0.598−0.841)	0.708 (0.572−0.845)
≤24 hours	0.901 (0.818−0.984)	0.880 (0.808−0.951)	0.893 (0.831−0.954)	0.918 (0.844−0.993)

cTnT: cardiac troponin T; SGCs: static growth charts; CGCs: conditional growth charts; VCMs: varying-coefficient models.

**Table 6.** The area under the ROC curve (AUC) and the 95% confidence interval for each of the historic approaches applied to the study dataset using leave-one-subject-out cross-validation and information up to time t .

	cTnT	COV-LDA	F-LDA	GFLM
≤6 hours	0.560 (0.432 − 0.689)	0.400 (0.263 − 0.537)	0.413 (0.282 − 0.544)	0.495 (0.374 − 0.615)
≤12 hours	0.712 (0.577 − 0.847)	0.537 (0.381 − 0.692)	0.597 (0.434 − 0.759)	0.677 (0.529 − 0.824)
≤24 hours	0.901 (0.818 − 0.984)	0.752 (0.584 − 0.920)	0.783 (0.625 − 0.941)	0.860 (0.737 − 0.983)

cTnT: cardiac troponin T; COV-LDA: covariance pattern longitudinal discriminant analysis; F-LDA: functional longitudinal discriminant analysis; GFLM: generalized functional linear model.

**Table 7.** The area under the ROC curve (AUC) and the 95% confidence interval of the maximum value for the study dataset using leave-one-subject-out cross-validation.

	Dynamic classification AUC
cTnT	0.901 (0.818 − 0.984)
SGC	0.880 (0.808 − 0.951)
CGC	0.893 (0.831 − 0.954)
VCM	0.918 (0.844 − 0.993)
COV-LDA	0.752 (0.584 − 0.920)
F-LDA	0.783 (0.625 − 0.941)
GFLM	0.860 (0.737 − 0.983)

cTnT: cardiac troponin-T; SGCs: static growth charts; CGCs: conditional growth charts; VCMs: varying-coefficient models; COV-LDA: covariance pattern longitudinal discriminant analysis; F-LDA: functional longitudinal discriminant analysis; GFLM: generalized functional linear model.

**Table 8.** Performance of different modeling approaches when defining a threshold equal to the sensitivity of the cTnT guideline.

	Sensitivity	Specificity	TP	FP	TN	FN	ARL
cTnT guideline	0.952	0.160	20	419	80	1	5.51
SGC	0.952	0.517	20	241	258	1	8.90
CGC	0.952	0.621	20	189	310	1	7.40
VCM	0.952	0.737	20	131	368	1	11.46
COV-LDA	0.952	0.028	20	485	14	1	8.66
F-LDA	0.952	0.072	20	463	36	1	1.71
GFLM	0.952	0.275	20	362	137	1	13.58

TP: true positives; FP: false positives; TN: true negatives; ARL: average run length; cTnT: cardiac troponin-T; SGCs: static growth charts; CGCs: conditional growth charts; VCMs: varying-coefficient models; COV-LDA: covariance pattern longitudinal discriminant analysis; F-LDA: functional longitudinal discriminant analysis; GFLM: generalized functional linear model.

## 7. Discussion

In this study, we described and compared several popular semi-parametric modeling approaches that combine irregularly and sparsely sampled measurements with a binary outcome. Our results show that functional regression models that implicitly incorporate historic information through the estimation of a covariance function, outperform models that do not incorporate historic information. The GFLM performed best of the approaches that incorporate historic information, while the VCM and static growth charts performed best of the approaches that do not incorporate historic information. The degree of sparsity has an effect on the ability of the functional regression approaches to outperform the non-historic approaches. Under settings with very high degrees of sparsity (down to a few measurements per subject) there is little benefit in incorporating historic information through (functional) random effects. The event rate of the outcome also has an impact on discriminative ability; lower event rates reduce discriminative ability, but this effect is equal across approaches. Except for settings with a high degree of sparsity and conditional growth charts, all modeling approaches show a benefit in terms of discriminative ability in a dynamic classification setting, compared to the clinical practice of using a fixed threshold on the raw measured value.

CGCs appear to be less suitable for (dynamic) classification of irregularly and sparsely sampled curves. In part, this is due to the fact that growth charts are not developed with classification in mind.^32^ The CGC approach, which explicitly incorporates historical information through AR terms, seems to offer a benefit in early detection of cases but not for later time points (see Figures 2 and 3). In this study, the CGC model as defined in (6) is referred to as a ‘‘global model” by Wei et al.^32^ This model is restrictive in the sense that it assumes that AR coefficients are linear functions of measurement time distances. Wei et al. describe several generalizations of the global model, for example, allowing the AR coefficients to be functions of measurement time distances. These generalizations could improve the performance of the CGC model. Moreover, in this example, an AR(1) model was used restricting historic information to the previous measurement only, including higher-order lags could improve performance. This also explains the counterintuitive finding depicted in Figure 2, where the CGC approach performs worse under *lower* degrees of sparsity versus *high* degrees of sparsity. With more frequent sampling, the previous (i.e. lag 1) measurement is closer in time, and therefore information from earlier measurements is ‘‘forgotten”, lowering the discriminative ability.

The functional regression approaches (F-LDA and GFLM) performed best on the simulated data. Although the F-LDA and GFLM approaches perform similar in this study, this may not always be the case. In a study by Hughes et al.^47^ that compared three different approaches to calculate a patient’s posterior group membership based on random effects, they concluded that the marginal approach (comparable to our F-LDA approach) performs best when the mean profile is noticeably different between groups. The GFLM approach could be a better option if the difference between the groups is characterized by the variability around the mean profile. The GFLM approach uses the principal component (PC) scores as covariates in a logistic regression model. However, using PC scores as predictors is not without downsides. With PC scores, there is no guarantee that the groups are separated best in the direction of the PC scores with the highest variance.^48^ In this study, we choose PC scores based on the percentage variance explained, but an alternative approach could be to apply a variable selection technique to choose PC scores based on their ability to separate groups. The F-LDA also outperformed the COV-LDA approach, this could be a result of the continuous time AR(1) correlation structure being too simple to effectively model the data. Different correlation structures could improve the performance of the COV-LDA approach, as well as incorporating class-specific correlation structures.

In different simulation scenarios, we investigated the (potential) effect of the degree of sparsity and event rates on the performance of the dynamic longitudinal classification approaches. When comparing high versus low degrees of sparsity (the high scenario corresponds to an average of four measurements per subject and the low scenario to 12 measurements per subject), the functional regression approaches suffer the most in terms of discriminative ability. This is not surprising, as there is less historic information available to estimate random effects in a sparse setting. However, this is not to say that the functional approaches offer no benefit in very sparse situations, but rather that it takes longer for this benefit to appear in a longitudinal setting. As can be seen in Figure 2, the functional approaches outperform the other approaches at t=18 versus t=8 in the case of high versus low sparsity. When comparing high versus low outcome event rates, there is no obvious difference in effect on the different approaches. In case of class imbalance, a low event rate results in reduced performance and more variability compared to a high event rate. This is also not unexpected, as there is less information to discriminate cases from controls, but this affects all approaches equally.

The GFLM and F-LDA approaches did not outperform the growth chart and VCM approaches in the clinical case study. However, this does not invalidate the conclusions from the simulations for several reasons. First, the clinical case study is comparable in the number of measurements per subject to the simulated data with a high degree of sparsity. Therefore, approaches based on historic information are more affected in terms of predictive performance, as can also be seen in the simulation results (Table 1). Second, while the clinical case study is comparable to the high degree of sparsity in terms of measurements per subject, the sparsity in the clinical case study is not evenly distributed over the time interval. There is more dense sampling at t<6 hours, and more sparse sampling at t>12 hours. This can have a negative impact on the estimation of the covariance function in the GFLM and F-LDA approaches. Finally, in part due to the smaller sample size, there is overlap in the AUC confidence intervals. We expect that with more frequent and evenly distributed sampling, the GFLM and F-LDA approaches are capable of outperforming the growth chart and VCM approaches. For further research, it would be interesting to take into account not only the degree of sparsity, but also the distribution of sampling times to assess the effect of uneven sampling distributions on, for example, covariance function estimation.

## 8. Copyright statement

Please be aware that the use of this LATE X2ɛ class file is governed by the following conditions.

### 8.1. Copyright

Copyright © 2023 SAGE Publications Ltd, 1 Oliver’s Yard, 55 City Road, London, EC1Y 1SP, UK. All rights reserved.

### 8.2. Rules of use

This class file is made available for use by authors who wish to prepare an article for publication in a *SAGE Publications* journal. The user may not exploit any part of the class file commercially.

This class file is provided on an *as is* basis, without warranties of any kind, either express or implied, including but not limited to warranties of title, or implied warranties of merchantablility or fitness for a particular purpose. There will be no duty on the author[s] of the software or SAGE Publications Ltd to correct any errors or defects in the software. Any statutory rights you may have remain unaffected by your acceptance of these rules of use.

## Supplemental Material

sj-pdf-1-smm-10.1177_09622802251374288 - Supplemental material for A comparison of semi-parametric statistical modeling approaches to dynamic classification of irregularly and sparsely sampled curves Supplemental material, sj-pdf-1-smm-10.1177_09622802251374288 for A comparison of semi-parametric statistical modeling approaches to dynamic classification of irregularly and sparsely sampled curves by Ruben Deneer, Zhuozhao Zhan, Edwin Van den Heuvel, Astrid GM van Boxtel, Arjen-Kars Boer, Natal AW van Riel and Volkher Scharnhorst in Statistical Methods in Medical Research

sj-pdf-2-smm-10.1177_09622802251374288 - Supplemental material for A comparison of semi-parametric statistical modeling approaches to dynamic classification of irregularly and sparsely sampled curves Supplemental material, sj-pdf-2-smm-10.1177_09622802251374288 for A comparison of semi-parametric statistical modeling approaches to dynamic classification of irregularly and sparsely sampled curves by Ruben Deneer, Zhuozhao Zhan, Edwin Van den Heuvel, Astrid GM van Boxtel, Arjen-Kars Boer, Natal AW van Riel and Volkher Scharnhorst in Statistical Methods in Medical Research
