# Supplementary material for: A comparison of semi-parametric statistical modeling approaches to dynamic classification of irregularly and sparsely sampled curves
Source: Stat Methods Med Res. 2025 Sep 4;34(11):2202–18. doi: 10.1177/09622802251374288 (PMC12669398; doi:10.1177/09622802251374288)
Supplement: sj-pdf-1-smm-10.1177_09622802251374288 - Supplemental material for A comparison of semi-parametric statistical modeling approaches to dynamic classification of irregularly and sparsely sampled curves [file sj-pdf-1-smm-10.1177_09622802251374288.pdf]

Supplemental Material

In the supplemental material the results of several different simulation scenarios are given. The scenarios differ in the degree of sparsity (missingness rate) and the proportion of patients with the outcome (event rate). SCG = static growth charts, CGC = conditional growth charts, VCM = varying coefficient models, COV-LDA = covariance pattern longitudinal discriminant analysis, GFLM = generalized functional linear model, F-LDA = functional longitudinal discriminant analysis.

Varying degrees of sparsity and outcome event rates

j	Raw value	SGC	CGC	VCM	GFLM	COV-LDA	F-LDA
1	0.462 (0.006)	0.462 (0.006)	NA (NA)	0.505 (0.007)	0.492 (0.008)	**0.518 (0.008)**	0.505 (0.008)
2	0.523 (0.006)	0.523 (0.006)	**0.550 (0.006)**	0.529 (0.007)	0.487 (0.008)	0.521 (0.008)	0.494 (0.008)
3	0.606 (0.005)	0.607 (0.005)	**0.648 (0.006)**	0.604 (0.006)	0.564 (0.010)	0.632 (0.007)	0.628 (0.007)
4	0.676 (0.006)	0.676 (0.006)	0.682 (0.005)	0.674 (0.006)	0.681 (0.008)	0.709 (0.008)	**0.721 (0.006)**
5	0.733 (0.005)	0.733 (0.005)	0.702 (0.005)	0.732 (0.005)	0.784 (0.005)	0.763 (0.007)	**0.795 (0.005)**
6	0.783 (0.005)	0.782 (0.005)	0.715 (0.006)	0.783 (0.005)	0.849 (0.004)	0.806 (0.007)	**0.850 (0.004)**
7	0.818 (0.004)	0.818 (0.004)	0.720 (0.004)	0.819 (0.005)	**0.889 (0.004)**	0.835 (0.006)	0.888 (0.004)
8	0.851 (0.004)	0.850 (0.004)	0.738 (0.005)	0.852 (0.004)	0.917 (0.003)	0.860 (0.005)	**0.918 (0.003)**
9	0.877 (0.003)	0.875 (0.003)	0.748 (0.005)	0.877 (0.003)	0.938 (0.003)	0.880 (0.005)	**0.941 (0.003)**
10	0.898 (0.003)	0.897 (0.003)	0.757 (0.005)	0.899 (0.003)	0.952 (0.003)	0.897 (0.005)	**0.958 (0.003)**
11	0.915 (0.003)	0.913 (0.003)	0.771 (0.005)	0.916 (0.003)	0.962 (0.003)	0.911 (0.004)	**0.971 (0.002)**
12	0.930 (0.003)	0.929 (0.003)	0.783 (0.005)	0.931 (0.003)	0.969 (0.002)	0.923 (0.004)	**0.980 (0.002)**
13	0.943 (0.003)	0.941 (0.003)	0.795 (0.006)	0.944 (0.003)	0.974 (0.002)	0.934 (0.004)	**0.986 (0.002)**

Table 1. Dynamic classification performance as expressed in AUC for each approach at each occasion j with the standard error in round brackets and the maximum in bold. Proportion of missing values (sparsity): 0%, proportion of patients with outcome (event rate): 10%

	AUC
Raw value	0.867 (0.004)
SGC	0.900 (0.003)
CGC	0.834 (0.004)
VCM	0.897 (0.005)
GFLM	0.953 (0.003)
COV-LDA	0.873 (0.006)
F-LDA	**0.957 (0.003)**

Table 2. The AUC of the maximum value in the time interval $\mathcal{T}$ for each approach and the maximum in bold. Proportion of missing values (sparsity): 0%, proportion of patients with outcome (event rate): 10%

j	Raw value	SGC	CGC	VCM	GFLM	COV-LDA	F-LDA
1	0.472 (0.006)	0.472 (0.006)	NA (NA)	0.512 (0.006)	0.520 (0.007)	**0.527 (0.006)**	0.520 (0.007)
2	0.509 (0.007)	0.509 (0.007)	**0.531 (0.006)**	0.520 (0.007)	0.504 (0.007)	0.528 (0.006)	0.509 (0.008)
3	0.591 (0.006)	0.590 (0.006)	0.620 (0.005)	0.587 (0.007)	0.582 (0.008)	0.621 (0.006)	**0.623 (0.006)**
4	0.668 (0.005)	0.668 (0.005)	0.680 (0.007)	0.667 (0.006)	0.695 (0.008)	0.707 (0.007)	**0.726 (0.006)**
5	0.723 (0.006)	0.723 (0.006)	0.704 (0.007)	0.722 (0.006)	0.777 (0.007)	0.758 (0.006)	**0.793 (0.005)**
6	0.779 (0.005)	0.779 (0.005)	0.745 (0.006)	0.779 (0.005)	0.846 (0.004)	0.804 (0.005)	**0.847 (0.005)**
7	0.816 (0.005)	0.815 (0.005)	0.740 (0.005)	0.817 (0.005)	**0.888 (0.004)**	0.834 (0.006)	0.887 (0.004)
8	0.849 (0.004)	0.848 (0.004)	0.760 (0.006)	0.849 (0.004)	**0.916 (0.003)**	0.861 (0.005)	0.914 (0.003)
9	0.873 (0.004)	0.872 (0.004)	0.769 (0.005)	0.874 (0.004)	**0.938 (0.003)**	0.881 (0.005)	0.938 (0.003)
10	0.898 (0.004)	0.897 (0.004)	0.787 (0.005)	0.898 (0.004)	0.956 (0.003)	0.899 (0.004)	**0.959 (0.002)**
11	0.916 (0.004)	0.915 (0.004)	0.806 (0.005)	0.916 (0.004)	0.964 (0.002)	0.913 (0.004)	**0.969 (0.002)**
12	0.933 (0.004)	0.932 (0.004)	0.818 (0.006)	0.934 (0.004)	0.971 (0.002)	0.927 (0.004)	**0.978 (0.002)**
13	0.942 (0.003)	0.940 (0.003)	0.830 (0.006)	0.942 (0.003)	0.976 (0.002)	0.933 (0.004)	**0.983 (0.002)**

Table 3. Dynamic classification performance as expressed in AUC for each approach at each occasion j with the standard error in round brackets and the maximum in bold. Proportion of missing values (sparsity): 10%, proportion of patients with outcome (event rate): 10%

	AUC
Raw value	0.864 (0.004)
SGC	0.900 (0.004)
CGC	0.859 (0.004)
VCM	0.893 (0.005)
GFLM	**0.958 (0.003)**
COV-LDA	0.873 (0.006)
F-LDA	0.956 (0.004)

Table 4. The AUC of the maximum value in the time interval $\mathcal{T}$ for each approach and the maximum in bold. Proportion of missing values (sparsity): 10%, proportion of patients with outcome (event rate): 10%

j	Raw value	SGC	CGC	VCM	GFLM	COV-LDA	F-LDA
1	0.468 (0.007)	0.468 (0.007)	NA (NA)	0.500 (0.008)	0.520 (0.008)	**0.521 (0.008)**	0.509 (0.008)
2	0.512 (0.008)	0.511 (0.008)	0.532 (0.007)	0.517 (0.008)	**0.532 (0.008)**	0.515 (0.008)	0.498 (0.008)
3	0.596 (0.008)	0.596 (0.008)	0.605 (0.007)	0.599 (0.007)	0.604 (0.010)	**0.625 (0.008)**	0.620 (0.008)
4	0.671 (0.008)	0.671 (0.008)	0.674 (0.008)	0.673 (0.009)	0.694 (0.010)	0.703 (0.008)	**0.716 (0.008)**
5	0.723 (0.008)	0.723 (0.008)	0.715 (0.006)	0.725 (0.008)	0.766 (0.008)	0.755 (0.009)	**0.779 (0.007)**
6	0.770 (0.006)	0.769 (0.006)	0.754 (0.006)	0.775 (0.007)	0.830 (0.005)	0.796 (0.006)	**0.833 (0.005)**
7	0.811 (0.007)	0.811 (0.007)	0.779 (0.007)	0.814 (0.006)	**0.880 (0.005)**	0.835 (0.007)	0.876 (0.006)
8	0.840 (0.006)	0.840 (0.006)	0.788 (0.006)	0.843 (0.006)	**0.907 (0.004)**	0.856 (0.006)	0.904 (0.005)
9	0.869 (0.006)	0.867 (0.006)	0.819 (0.007)	0.869 (0.006)	**0.934 (0.004)**	0.880 (0.006)	0.931 (0.004)
10	0.889 (0.005)	0.888 (0.005)	0.831 (0.006)	0.892 (0.005)	**0.950 (0.004)**	0.894 (0.005)	0.949 (0.003)
11	0.913 (0.004)	0.911 (0.004)	0.850 (0.005)	0.915 (0.004)	0.963 (0.003)	0.911 (0.005)	**0.965 (0.003)**
12	0.927 (0.004)	0.925 (0.004)	0.861 (0.005)	0.928 (0.004)	0.972 (0.003)	0.924 (0.005)	**0.975 (0.002)**
13	0.940 (0.004)	0.938 (0.004)	0.878 (0.005)	0.942 (0.003)	0.976 (0.003)	0.932 (0.004)	**0.982 (0.002)**

Table 5. Dynamic classification performance as expressed in AUC for each approach at each occasion j with the standard error in round brackets and the maximum in bold. Proportion of missing values (sparsity): 25%, proportion of patients with outcome (event rate): 10%

	AUC
Raw value	0.857 (0.005)
SGC	0.891 (0.004)
CGC	0.861 (0.005)
VCM	0.887 (0.005)
GFLM	**0.957 (0.003)**
COV-LDA	0.873 (0.006)
F-LDA	0.940 (0.004)

Table 6. The AUC of the maximum value in the time interval $\mathcal{T}$ for each approach and the maximum in bold. Proportion of missing values (sparsity): 25%, proportion of patients with outcome (event rate): 10%

j	Raw value	SGC	CGC	VCM	GFLM	COV-LDA	F-LDA
1	0.473 (0.006)	0.473 (0.006)	NA (NA)	0.508 (0.005)	**0.513 (0.007)**	0.508 (0.007)	0.497 (0.007)
2	0.508 (0.010)	0.508 (0.009)	**0.529 (0.008)**	0.516 (0.010)	0.512 (0.010)	0.500 (0.010)	0.486 (0.010)
3	0.593 (0.008)	0.594 (0.007)	0.596 (0.009)	0.588 (0.008)	0.562 (0.010)	**0.604 (0.010)**	0.591 (0.010)
4	0.668 (0.008)	0.668 (0.008)	0.659 (0.007)	0.663 (0.008)	0.681 (0.010)	0.681 (0.011)	**0.689 (0.009)**
5	0.729 (0.007)	0.729 (0.007)	0.718 (0.008)	0.729 (0.007)	**0.761 (0.007)**	0.738 (0.009)	0.760 (0.008)
6	0.777 (0.007)	0.776 (0.007)	0.778 (0.006)	0.777 (0.007)	**0.819 (0.006)**	0.783 (0.009)	0.814 (0.007)
7	0.812 (0.007)	0.812 (0.007)	0.812 (0.007)	0.812 (0.007)	**0.863 (0.006)**	0.820 (0.007)	0.859 (0.006)
8	0.846 (0.007)	0.846 (0.007)	0.847 (0.006)	0.847 (0.007)	**0.895 (0.005)**	0.849 (0.006)	0.892 (0.005)
9	0.868 (0.006)	0.867 (0.006)	0.861 (0.006)	0.869 (0.005)	**0.923 (0.004)**	0.867 (0.007)	0.919 (0.005)
10	0.896 (0.005)	0.894 (0.005)	0.885 (0.006)	0.895 (0.005)	**0.945 (0.004)**	0.892 (0.005)	0.944 (0.004)
11	0.917 (0.004)	0.915 (0.004)	0.908 (0.004)	0.918 (0.004)	0.958 (0.003)	0.907 (0.005)	**0.960 (0.003)**
12	0.927 (0.004)	0.926 (0.004)	0.913 (0.005)	0.927 (0.004)	0.967 (0.003)	0.918 (0.005)	**0.971 (0.003)**
13	0.938 (0.004)	0.935 (0.004)	0.928 (0.004)	0.939 (0.004)	0.969 (0.004)	0.925 (0.004)	**0.976 (0.003)**

Table 7. Dynamic classification performance as expressed in AUC for each approach at each occasion j with the standard error in round brackets and the maximum in bold. Proportion of missing values (sparsity): 50%, proportion of patients with outcome (event rate): 10%

	AUC
Raw value	0.860 (0.004)
SGC	0.886 (0.004)
CGC	0.872 (0.004)
VCM	0.870 (0.005)
GFLM	**0.945 (0.003)**
COV-LDA	0.854 (0.006)
F-LDA	0.920 (0.004)

Table 8. The AUC of the maximum value in the time interval $\mathcal{T}$ for each approach and the maximum in bold. Proportion of missing values (sparsity): 50%, proportion of patients with outcome (event rate): 10%

j	Raw value	SGC	CGC	VCM	GFLM	COV-LDA	F-LDA
1	0.464 (0.007)	0.465 (0.007)	NA (NA)	0.509 (0.007)	**0.527 (0.007)**	0.525 (0.008)	0.515 (0.008)
2	0.516 (0.015)	0.516 (0.015)	0.530 (0.014)	0.515 (0.016)	**0.541 (0.014)**	0.501 (0.015)	0.504 (0.015)
3	0.601 (0.015)	0.601 (0.015)	**0.611 (0.014)**	0.586 (0.015)	0.600 (0.014)	0.594 (0.016)	0.577 (0.017)
4	0.684 (0.013)	0.684 (0.013)	**0.692 (0.013)**	0.681 (0.013)	0.686 (0.014)	0.689 (0.015)	0.685 (0.015)
5	0.751 (0.010)	0.751 (0.010)	0.755 (0.011)	0.747 (0.010)	**0.765 (0.010)**	0.756 (0.010)	0.758 (0.010)
6	0.791 (0.010)	0.791 (0.010)	0.793 (0.010)	0.790 (0.011)	0.811 (0.010)	0.805 (0.011)	**0.817 (0.009)**
7	0.824 (0.009)	0.823 (0.009)	0.826 (0.008)	0.824 (0.009)	0.850 (0.008)	0.835 (0.009)	**0.853 (0.007)**
8	0.845 (0.009)	0.844 (0.009)	0.852 (0.009)	0.847 (0.008)	0.870 (0.008)	0.847 (0.010)	**0.870 (0.009)**
9	0.870 (0.009)	0.869 (0.009)	0.875 (0.008)	0.870 (0.009)	0.898 (0.008)	0.875 (0.009)	**0.901 (0.008)**
10	0.902 (0.006)	0.901 (0.006)	0.904 (0.006)	0.901 (0.007)	0.926 (0.007)	0.892 (0.009)	**0.937 (0.006)**
11	0.913 (0.007)	0.912 (0.007)	0.916 (0.006)	0.913 (0.007)	0.939 (0.006)	0.905 (0.007)	**0.948 (0.005)**
12	0.928 (0.006)	0.926 (0.006)	0.930 (0.006)	0.929 (0.006)	0.946 (0.006)	0.916 (0.007)	**0.963 (0.005)**
13	0.944 (0.005)	0.942 (0.004)	0.945 (0.004)	0.943 (0.005)	0.956 (0.005)	0.930 (0.005)	**0.971 (0.003)**

Table 9. Dynamic classification performance as expressed in AUC for each approach at each occasion j with the standard error in round brackets and the maximum in bold. Proportion of missing values (sparsity): 75%, proportion of patients with outcome (event rate): 10%

	AUC
Raw value	0.846 (0.005)
SGC	0.860 (0.005)
CGC	0.855 (0.005)
VCM	0.849 (0.005)
GFLM	**0.886 (0.006)**
COV-LDA	0.839 (0.005)
F-LDA	0.875 (0.005)

Table 10. The AUC of the maximum value in the time interval $\mathcal{T}$ for each approach and the maximum in bold. Proportion of missing values (sparsity): 75%, proportion of patients with outcome (event rate): 10%

j	Raw value	SGC	CGC	VCM	GFLM	COV-LDA	F-LDA
1	0.466 (0.004)	0.466 (0.004)	NA (NA)	0.506 (0.005)	0.485 (0.006)	**0.520 (0.006)**	0.512 (0.006)
2	0.513 (0.004)	0.515 (0.004)	**0.538 (0.004)**	0.519 (0.003)	0.476 (0.005)	0.522 (0.006)	0.503 (0.006)
3	0.597 (0.004)	0.597 (0.004)	**0.643 (0.004)**	0.599 (0.004)	0.566 (0.008)	0.626 (0.004)	0.632 (0.004)
4	0.673 (0.004)	0.673 (0.004)	0.689 (0.004)	0.675 (0.004)	0.688 (0.006)	0.711 (0.005)	**0.732 (0.004)**
5	0.733 (0.004)	0.732 (0.004)	0.701 (0.004)	0.735 (0.004)	0.782 (0.005)	0.768 (0.005)	**0.805 (0.004)**
6	0.785 (0.003)	0.785 (0.003)	0.724 (0.003)	0.788 (0.003)	0.849 (0.004)	0.814 (0.004)	**0.860 (0.003)**
7	0.821 (0.003)	0.820 (0.003)	0.722 (0.004)	0.823 (0.003)	0.892 (0.003)	0.843 (0.004)	**0.897 (0.003)**
8	0.853 (0.003)	0.852 (0.003)	0.738 (0.004)	0.855 (0.003)	0.922 (0.002)	0.867 (0.004)	**0.925 (0.002)**
9	0.879 (0.002)	0.878 (0.002)	0.752 (0.003)	0.880 (0.002)	0.942 (0.002)	0.886 (0.003)	**0.947 (0.002)**
10	0.900 (0.002)	0.898 (0.002)	0.757 (0.004)	0.902 (0.002)	0.957 (0.002)	0.902 (0.003)	**0.962 (0.002)**
11	0.917 (0.002)	0.915 (0.002)	0.772 (0.004)	0.918 (0.002)	0.966 (0.001)	0.915 (0.003)	**0.973 (0.001)**
12	0.933 (0.002)	0.931 (0.002)	0.787 (0.003)	0.935 (0.002)	0.974 (0.001)	0.928 (0.002)	**0.982 (0.001)**
13	0.945 (0.002)	0.942 (0.002)	0.795 (0.004)	0.946 (0.002)	0.979 (0.001)	0.937 (0.002)	**0.987 (0.001)**

Table 11. Dynamic classification performance as expressed in AUC for each approach at each occasion j with the standard error in round brackets and the maximum in bold. Proportion of patients with outcome (event rate): 20%, no missing values

	AUC
Raw value	0.869 (0.002)
SGC	0.903 (0.002)
CGC	0.839 (0.003)
VCM	0.901 (0.003)
GFLM	0.945 (0.002)
COV-LDA	0.879 (0.004)
F-LDA	**0.961 (0.002)**

Table 12. The AUC of the maximum value in the time interval $\mathcal{T}$ for each approach and the maximum in bold. Proportion of patients with outcome (event rate): 20%, no missing values

j	Raw value	SGC	CGC	VCM	GFLM	COV-LDA	F-LDA
1	0.461 (0.006)	0.461 (0.006)	NA (NA)	0.501 (0.007)	0.483 (0.007)	**0.524 (0.007)**	0.511 (0.008)
2	0.515 (0.008)	0.514 (0.008)	**0.543 (0.007)**	0.517 (0.009)	0.482 (0.008)	0.533 (0.009)	0.498 (0.009)
3	0.602 (0.008)	0.602 (0.008)	**0.649 (0.006)**	0.605 (0.007)	0.554 (0.010)	0.644 (0.007)	0.635 (0.006)
4	0.675 (0.007)	0.675 (0.007)	0.686 (0.005)	0.678 (0.007)	0.681 (0.008)	0.726 (0.007)	**0.731 (0.005)**
5	0.730 (0.006)	0.730 (0.006)	0.695 (0.005)	0.730 (0.006)	0.781 (0.006)	0.772 (0.006)	**0.799 (0.005)**
6	0.777 (0.006)	0.777 (0.006)	0.710 (0.005)	0.777 (0.006)	0.843 (0.005)	0.812 (0.006)	**0.849 (0.005)**
7	0.816 (0.005)	0.816 (0.005)	0.727 (0.006)	0.816 (0.005)	0.888 (0.004)	0.842 (0.005)	**0.890 (0.004)**
8	0.847 (0.005)	0.846 (0.005)	0.730 (0.007)	0.848 (0.005)	0.918 (0.003)	0.865 (0.005)	**0.920 (0.003)**
9	0.873 (0.004)	0.872 (0.004)	0.747 (0.006)	0.873 (0.004)	0.937 (0.003)	0.884 (0.004)	**0.943 (0.002)**
10	0.896 (0.004)	0.895 (0.004)	0.758 (0.005)	0.896 (0.004)	0.953 (0.002)	0.901 (0.004)	**0.960 (0.002)**
11	0.914 (0.004)	0.913 (0.004)	0.768 (0.006)	0.915 (0.004)	0.963 (0.002)	0.913 (0.004)	**0.972 (0.002)**
12	0.929 (0.004)	0.927 (0.004)	0.774 (0.005)	0.930 (0.003)	0.971 (0.002)	0.924 (0.004)	**0.980 (0.002)**
13	0.941 (0.003)	0.939 (0.003)	0.792 (0.006)	0.941 (0.003)	0.976 (0.002)	0.934 (0.003)	**0.986 (0.001)**

Table 13. Dynamic classification performance as expressed in AUC for each approach at each occasion j with the standard error in round brackets and the maximum in bold. Proportion of patients with outcome (event rate): 10%, no missing values

	AUC
Raw value	0.863 (0.005)
SGC	0.898 (0.004)
CGC	0.834 (0.005)
VCM	0.899 (0.005)
GFLM	0.954 (0.002)
COV-LDA	0.880 (0.005)
F-LDA	**0.961 (0.002)**

Table 14. The AUC of the maximum value in the time interval $\mathcal{T}$ for each approach and the maximum in bold. Proportion of patients with outcome (event rate): 10%, no missing values

j	Raw value	SGC	CGC	VCM	GFLM	COV-LDA	F-LDA
1	0.475 (0.009)	0.475 (0.009)	NA (NA)	0.508 (0.011)	0.493 (0.010)	**0.521 (0.010)**	0.518 (0.010)
2	0.503 (0.009)	0.503 (0.009)	**0.524 (0.009)**	0.504 (0.010)	0.488 (0.008)	0.519 (0.009)	0.500 (0.010)
3	0.587 (0.009)	0.587 (0.009)	**0.645 (0.008)**	0.575 (0.011)	0.550 (0.010)	0.625 (0.009)	0.625 (0.009)
4	0.658 (0.009)	0.658 (0.009)	0.684 (0.008)	0.658 (0.009)	0.680 (0.009)	0.696 (0.008)	**0.725 (0.007)**
5	0.714 (0.008)	0.714 (0.008)	0.688 (0.009)	0.712 (0.008)	0.773 (0.007)	0.743 (0.008)	**0.788 (0.006)**
6	0.765 (0.007)	0.765 (0.007)	0.716 (0.007)	0.764 (0.008)	0.842 (0.006)	0.789 (0.008)	**0.843 (0.006)**
7	0.801 (0.006)	0.801 (0.006)	0.710 (0.007)	0.799 (0.007)	0.881 (0.005)	0.819 (0.006)	**0.881 (0.005)**
8	0.841 (0.006)	0.840 (0.006)	0.746 (0.007)	0.840 (0.006)	0.912 (0.004)	0.849 (0.006)	**0.916 (0.004)**
9	0.863 (0.006)	0.862 (0.006)	0.733 (0.007)	0.861 (0.006)	0.932 (0.004)	0.868 (0.005)	**0.940 (0.003)**
10	0.890 (0.005)	0.889 (0.005)	0.758 (0.007)	0.889 (0.005)	0.947 (0.003)	0.889 (0.005)	**0.957 (0.003)**
11	0.907 (0.005)	0.905 (0.005)	0.758 (0.007)	0.907 (0.005)	0.957 (0.003)	0.903 (0.005)	**0.969 (0.003)**
12	0.923 (0.004)	0.921 (0.004)	0.776 (0.008)	0.925 (0.004)	0.965 (0.003)	0.917 (0.004)	**0.978 (0.002)**
13	0.939 (0.004)	0.936 (0.004)	0.796 (0.005)	0.941 (0.003)	0.971 (0.003)	0.929 (0.004)	**0.984 (0.002)**

Table 15. Dynamic classification performance as expressed in AUC for each approach at each occasion j with the standard error in round brackets and the maximum in bold. Proportion of patients with outcome (event rate): 5%, no missing values

	AUC
Raw value	0.857 (0.005)
SGC	0.892 (0.004)
CGC	0.830 (0.005)
VCM	0.885 (0.006)
GFLM	**0.955 (0.003)**
COV-LDA	0.863 (0.008)
F-LDA	0.953 (0.004)

Table 16. The AUC of the maximum value in the time interval $\mathcal{T}$ for each approach and the maximum in bold. Proportion of patients with outcome (event rate): 5%, no missing values

j	Raw value	SGC	CGC	VCM	GFLM	COV-LDA	F-LDA
1	0.455 (0.013)	0.455 (0.013)	NA (NA)	0.535 (0.012)	0.486 (0.015)	**0.541 (0.014)**	0.508 (0.015)
2	0.505 (0.012)	0.506 (0.012)	**0.536 (0.012)**	0.509 (0.013)	0.484 (0.014)	0.506 (0.013)	0.493 (0.013)
3	0.589 (0.012)	0.588 (0.012)	**0.642 (0.010)**	0.580 (0.013)	0.526 (0.015)	0.599 (0.013)	0.602 (0.014)
4	0.656 (0.012)	0.656 (0.012)	0.668 (0.011)	0.651 (0.013)	0.640 (0.014)	0.664 (0.012)	**0.702 (0.011)**
5	0.712 (0.011)	0.711 (0.011)	0.682 (0.011)	0.709 (0.011)	0.752 (0.011)	0.715 (0.012)	**0.775 (0.010)**
6	0.756 (0.011)	0.756 (0.011)	0.697 (0.011)	0.756 (0.011)	0.819 (0.010)	0.754 (0.012)	**0.824 (0.011)**
7	0.795 (0.010)	0.794 (0.010)	0.723 (0.008)	0.793 (0.010)	**0.866 (0.008)**	0.791 (0.011)	0.864 (0.009)
8	0.828 (0.009)	0.827 (0.009)	0.719 (0.010)	0.826 (0.009)	0.894 (0.007)	0.819 (0.010)	**0.897 (0.007)**
9	0.855 (0.009)	0.854 (0.009)	0.732 (0.010)	0.850 (0.010)	0.917 (0.006)	0.844 (0.009)	**0.924 (0.006)**
10	0.885 (0.008)	0.883 (0.008)	0.759 (0.011)	0.882 (0.008)	0.935 (0.006)	0.871 (0.008)	**0.948 (0.005)**
11	0.901 (0.008)	0.899 (0.008)	0.756 (0.011)	0.898 (0.008)	0.948 (0.005)	0.887 (0.008)	**0.963 (0.004)**
12	0.918 (0.007)	0.916 (0.007)	0.765 (0.010)	0.915 (0.008)	0.958 (0.005)	0.902 (0.007)	**0.975 (0.003)**
13	0.935 (0.006)	0.932 (0.006)	0.790 (0.009)	0.931 (0.007)	0.966 (0.004)	0.916 (0.007)	**0.984 (0.003)**

Table 17. Dynamic classification performance as expressed in AUC for each approach at each occasion j with the standard error in round brackets and the maximum in bold. Proportion of patients with outcome (event rate): 2.5%, no missing values

	AUC
Raw value	0.849 (0.009)
SGC	0.887 (0.008)
CGC	0.830 (0.008)
VCM	0.873 (0.011)
GFLM	0.952 (0.005)
COV-LDA	0.839 (0.011)
F-LDA	**0.954 (0.006)**

Table 18. The AUC of the maximum value in the time interval $\mathcal{T}$ for each approach and the maximum in bold. Proportion of patients with outcome (event rate): 2.5%, no missing values
