# Supplementary material for: A comparison of semi-parametric statistical modeling approaches to dynamic classification of irregularly and sparsely sampled curves
Source: Stat Methods Med Res. 2025 Sep 4;34(11):2202–18. doi: 10.1177/09622802251374288 (PMC12669398; doi:10.1177/09622802251374288)
Supplement: sj-pdf-2-smm-10.1177_09622802251374288 - Supplemental material for A comparison of semi-parametric statistical modeling approaches to dynamic classification of irregularly and sparsely sampled curves [file sj-pdf-2-smm-10.1177_09622802251374288.pdf]

Supplemental material - R code

In this supplemental material we provide code to fit the different approaches described in the paper "*A comparison of semi-parametric statistical modeling approaches to dynamic classification of irregularly and sparsely sampled curves.*". The `data` `sim_w_resid` are the simulated biomarker values, `sample_time` are the (simulated) sampling times of the biomarker values, `PMI` is a logical which is TRUE if the patient belongs to the PMI class and FALSE otherwise.

Growth charts

Below are the functions used to fit static growth charts (`fit.static.gc`), fit conditional growth charts (`fit.cond.gc`) and to find the quantile to which a measurement belongs (`transform.to.q`). `qu_vec` is a grid vector of quantiles fitted by the `qgam` library.

```
library(qgam)

# Fit smooth additive quantile regression model to non-PMI patients
fit.static.gc <- function(data, qu_vec){
  mqgam(sim_w_resid ~ s(sample_time, bs = "ps"),
        data = subset(data, !PMI), qu = qu_vec, multicore = FALSE)
}

# Fit smooth additive quantile regression model to non-PMI patients
fit.cond.gc <- function(data, qu_vec){
  mqgam(sim_w_resid ~ s(sample_time, bs = "ps") +
        sim_w_resid_lag1 + sim_w_resid_lag1:time_gap1,
        data = subset(data, !PMI), qu = qu_vec, multicore = FALSE)
}

# Convert measurements to quantile interval
transform.to.q <- function(data, mqgam_fit){
  # Fitted quantiles
  q_vec <- names(mqgam_fit$fit)
  preds <- qdo(obj = mqgam_fit, fun = predict, newdata = data)
  qus <- do.call(cbind, preds)
  # Convert to ordered factor with quantile interval
  # that contains the measurement
  qus_bin <- data$sim_w_resid >= qus
  rsum <- rowSums(qus_bin)
  labels <- levels(cut(as.numeric(q_vec), c(0, q_vec, 1)))
  ordered(labels[rsum + 1], levels = labels)
}
```

Varying-coefficient model

Below is the function to fit the varying-coefficient model (VCM).

```
library(mgcv)

# Model with a tensor product interaction between the simulated troponin value
# and the time.
fit.tensor.product <- function(data){
```

```r
  gam(PMI ~ te(sim_w_resid, sample_time, bs = "ps", k = 8),
      data = data.frame(data), family = binomial())
}
```

Generalized functional linear model

Below are the functions to fit the generalized functional linear model (`fit.two.stage`) and to predict the probability that a new subject belongs to the PMI class (`predict.two.stage`). Also, a convenience function was created which predicts probabilities sequentially for a new subject (`predict.two.stage.seq`).

```r
library(face)
library(mgcv)
library(doParallel)


# Two stage implementation -----------------------------------------------

fit.two.stage <- function(data, pred.times = NULL){
  gam.mod <- gam(sim_w_resid ~ s(sample_time, bs = "ps"), data = data)
  gam.res <- resid(gam.mod)
  dat <- with(data, data.frame(argvals = sample_time,
                               subj = as.character(id),
                               y = gam.res))
  face.mod <- face.sparse(dat, newdata = dat, pve = 0.95,
                          argvals.new = pred.times, calculate.scores = T,
                          center = F)
  # Extract BLUPS and bind with outcome
  glmdat <- data.frame(score = face.mod$rand_eff$scores,
                       PMI = subset(data, !duplicated(id), select = 'PMI'))
  # Fit logistic regression model with BLUPS as covariates
  glm.mod <- glm(PMI ~ ., data = glmdat, family = binomial())
  list(gam_model = gam.mod, face_model = face.mod, logit_mod = glm.mod)
}


# Function to predict for new subjects based on observed data
predict.two.stage <- function(gam.model, face.model, logit.model, subject.data,
                              t.max = Inf, return.predict.face.obj = FALSE){
  # New data up to time t_max (if defined)
  subject.data <- subset(subject.data, !is.na(sim_w_resid) & time <= t.max)
  mu <- predict(gam.model, subject.data)
  x <- subject.data$sim_w_resid - mu
  # New data for predict function, note that this is only used to get
  # the covariance matrix (not to obtain scores), therefore y is not used
  face_datnew <- data.frame(argvals = subject.data$sample_time,
                            subj = as.character("99999999"),
                            y = x)
  # Predict and extract E-BLUPS
  face.preds <- predict(face.model, face_datnew)
  face.eblups <- data.frame(score = face.preds$rand_eff$scores)
  # Plug EBLUPS in GLM model to obtain probability of PMI
  p <- predict(logit.model, face.eblups, type = "response")
```

```
  if (return.predict.face.obj) {
    list(predict.face.obj = face.preds, prob = p)
  } else {
    p
  }
}

# Function to predict two stage sequentially
predict.two.stage.seq <- function(gam.model, face.model, logit.model,
                                  subject.data){
  times <- subject.data$time
  sapply(times, predict.two.stage, gam.model = gam.model,
         face.model = face.model, logit.model = logit.model,
         subject.data = subject.data)
}
```

Covariance pattern LDA

Below are the functions to fit the covariance pattern longitudinal discriminant analysis (`fit.gamm.CAR1`) and to predict the probability that a new subject belongs to the PMI class (`predict.gamm.CAR1`). Also, a convenience function was created which predicts probabilities sequentially for a new subject (`predict.gamm.CAR1.seq`).

```
library(mgcv)
library(mvtnorm)


# LongDA -----------------------------------------------------------------


fit.gamm.CAR1 <- function(data){
  gamm(sim_w_resid ~ factor(PMI) + s(sample_time, by = factor(PMI), bs = "ps"),
       correlation = corCAR1(form = ~ sample_time | id),
       data = data, method = "REML")
}

predict.gamm.CAR1 <- function(gamm_fit, subject_data, t_max = Inf){
  # New data up to time t_max (if defined)
  datnew <- subset(subject_data, !is.na(sim_w_resid) & time <= t_max)
  Y <- datnew$sim_w_resid
  x <- datnew$sample_time
  # Correlation structure from model
  cS <- gamm_fit$lme$modelStruct$corStruct
  # Calculate variance covariance matrix D, for new subject
  R <- corMatrix(cS, covariate = x)
  s <- sigma(gamm_fit$lme)
  D <- diag(s, dim(R)) %*% R %*% diag(s, dim(R))
  # Class means
  mu_PMI <- predict(gamm_fit$gam, data.frame(sample_time = x, PMI = T))
  mu_no_PMI <- predict(gamm_fit$gam, data.frame(sample_time = x, PMI = F))
  # Priors
  pi_pmi <- mean(gamm_fit$gam$model$`factor(PMI)` == "TRUE")
```

```r
  pi_no_pmi <- 1 - pi_pmi
  # Multivariate normal density
  f_PMI <- dmvnorm(Y, mean = mu_PMI, sigma = D)
  f_no_PMI <- dmvnorm(Y, mean = mu_no_PMI, sigma = D)
  # Bayes rule
  f_PMI*pi_pmi/(f_PMI*pi_pmi + f_no_PMI*pi_no_pmi)
}

# Function to predict sequentially
predict.gamm.CAR1.seq <- function(gamm_fit, subject_data){
  times <- subject_data$time
  sapply(times, predict.gamm.CAR1, gamm_fit = gamm_fit,
         subject_data = subject_data)
}
```

Functional LDA

Below are the functions to fit the covariance pattern longitudinal discriminant analysis (`fit.flda.face`) and to predict the probability that a new subject belongs to the PMI class (`predict.flda.face`). Also, a convenience function was created which predicts probabilities sequentially for a new subject (`predict.flda.face.seq`).

```r
# Load
library(face)
library(mgcv)
library(mvtnorm)

# LongDA -----------------------------------------------------------------

fit.flda.face <- function(data, pred.times = NULL){
  gam.mod <- gam(sim_w_resid ~ factor(PMI) +
                   s(sample_time, by = factor(PMI), bs = "ps"), data = data)
  gam.res <- resid(gam.mod)
  dat <- (with(data, data.frame(argvals = sample_time,
                                 subj = as.character(id),
                                 y = gam.res)))
  face.mod <- face.sparse(dat, newdata = dat, knots = 12, pve = 0.95,
                          argvals.new = pred.times, calculate.scores = F,
                          center = F)
  list(gam.mod = gam.mod, face.mod = face.mod)
}

# Function to predict for new subjects based on observed data
predict.flda.face <- function(gam.model, face.model, subject.data,
                              return.predict.face.obj = FALSE){
  # New data up to time t_max (if defined)
  subject.data <- subset(subject.data, !is.na(sim_w_resid))
  dataNoPMI <- subset(subject.data, select = -c(PMI))
  mu_PMI <- predict(gam.model, data.frame(dataNoPMI, 'PMI' = T))
  mu_no_PMI <- predict(gam.model, data.frame(dataNoPMI, 'PMI' = F))
  x_PMI <- subject.data$sim_w_resid - mu_PMI
  x_no_PMI <- subject.data$sim_w_resid - mu_no_PMI
  # New data for predict function, note that this is only used to get
```

```r
  # the covariance matrix (not to obtain scores), therefore y is not used
  face_datnew <- data.frame(argvals = subject.data$sample_time,
                            subj = as.character("99999999"),
                            y = x_no_PMI)
  face_pred <- predict(face.model, face_datnew)
  if (nrow(face_datnew) == 1) {
    D <- with(face_pred, Chat.pred + var.error.pred)
  } else {
    D <- with(face_pred, Chat.pred + diag(var.error.pred))
  }
  f_PMI <- dmvnorm(as.vector(x_PMI), sigma = D)
  f_no_PMI <- dmvnorm(as.vector(x_no_PMI), sigma = D)
  pi_pmi <- mean(gam.model$model$`factor(PMI)` == "TRUE")
  pi_no_pmi <- 1 - pi_pmi
  p <- f_PMI*pi_pmi/(f_PMI*pi_pmi + f_no_PMI*pi_no_pmi)
  if (return.predict.face.obj) {
    list(predict.face.obj = face_pred, prob = p)
  } else {
    p
  }
}

# Function to predict sequentially
predict.flda.face.seq <- function(flda.fit, subject_data){
  p <- vector(mode = "numeric", length = nrow(subject_data))
  subject_data <- data.frame(subject_data)
  for (i in 1:nrow(subject_data)) {
    p[i] <- predict.flda.face(gam.model = flda.fit$gam.mod,
                              face.model = flda.fit$face.mod,
                              subject.data = subject_data[1:i,])
  }
  p
}
```

Time-dependent AUC

A wrapper function was created around the `roc` function from the `pROC` package to obtain the AUC. The `input` argument are the predicted quantiles/probabilities on the test set, and the `outcome` argument the class labels (TRUE/FALSE) of the test set.

```r
library(pROC)

# pROC function which returns the AUC
get.auc <- function(outcome, input){
  if (all(is.na(input)) | all(input == -Inf)) return(as.numeric(NA))
  roc(response = outcome, predictor = as.numeric(input),
      levels = c(T, F), direction = ">")$auc
}
```

To obtain the dynamic classification AUC, the cumulative maximum is calculated for each patient using the `cummax.fun` and these cumulative maxima are then used to calculate the AUC by time. The `merged_bind` data.table contains the merged results of all the predictions on the test sets, with the predicted quantiles/probabilities of the different approaches in individual columns.

```r
# Function to get cumulative maximum that skips NAs
cummax.fun <- function(x){
  x <- as.numeric(x)
  cummax(ifelse(is.na(x), -Inf, x))
}

cummax_by_id <- lapply(merged_bind, function(x)
                        x[i > 0, c(lapply(.SD, cummax.fun),
                                   .(PMI = PMI[1], split = split[1], i = i)),
                          .SDcols = cols, by = .(split, id)])

auc_by_time_cummax <- lapply(cummax_by_id, function(x)
                             x[, lapply(.SD, get.auc, outcome = PMI),
                               .SDcols = cols, by = .(split, i)])
```
